# The Southern Medical and Surgical Journal

**Published:** 1851-08

**Authors:** 


### THE WESTERN JOURNAL.

THIRD SERIES.—VOL. VIII.—NO. II.

LOUISVILLE, AUGUST 1, 1851.

### THE SOUTHERN MEDICAL AND SURGICAL JOURNAL.

When a professional writer is so hard run as to attempt to answer a medical criticism by vulgar personalities, he betrays the weakness of the cause he advocates, and shows the inherent tendencies of his mind. In the April number of this Journal a case of lithotrity was quoted in full from the Southern Medical and Surgical Journal, and a series of remarks was appended to the quotation. There were great difficulties, in our judgment, in many of the opinions advanced, and we expressed those difficulties in respectful language. Although we had the assurance of able and experienced lithotritists that there were claims in the case, as reported, that were impossible, we did not say so in our publication. If this case stands the test of time, and proves the perfectness of the cure, it should be regarded as one of the greatest triumphs of lithotrity over lithotomy. But we wait the test of time.

In performing what we felt to be a duty, in calling attention to the extraordinary features of the case reported by Professor Dugas, we have aroused Dr. F., of the Georgia school, to the display of a fit of anger, and he indulges himself in a strain of personalities that is unworthy his character as a christian, a physician, and professor. He makes no attempt to remove the difficulties of the case. The novel idea that a phymosis caused a dilatation of the ureters, to such an extent that a mulberry calculus, "about an inch in length," and half an inch in its short diameter, passed through the ureter without causing feeling of any kind, and that the urethra was not dilated at all by this state of things is so subversive of every principle of hydraulics with which we are acquainted, that we feel sorry Dr. F. did not have the "patience" to explain it. He says he can explain it, and we should feel under obligations to him for an explanation. The force of a flood is usually expended at the point of obstruction, not at the remotest point from the dam.

We have not space now to follow Dr. F. through all his personalities, but there are a few points to which we shall attend. While Dr. F. was looming largely on the subject of rights and privileges, we think he might have remembered the golden rule, a rule as important to medical editors as to any other class in the community.

And now to Dr. F.'s statements: First, Dr. F. infers from the surprise we expressed at the celerity of the case, that we intended to charge Dr. Dugas with falsehood. Our language can bear no such interpretation. The celerity we complained of was in the rapid manner in which the crushing was effected, and the patient pronounced well. The crushing was performed on the 12th and 13th days of February, and in a few weeks after the report of the case is announced in the Southern Medical and Surgical Journal. That there was too much rapidity in all this is the doctrine of all lithotritists. Malgaigne, one the highest authorities on such subjects, says: "the records of a case," (of lithotrity) "to be of value, should comprise at least one year after the patient's discharge." And Mr. Curling, of the London hospital, cautiously concludes in a recent case, after *seven months'* observation of it, that "no portion of the calculus had been left behind." If the ablest lithotritists thus acknowledgo that *they* require a period of seven months or a year, in order to determine whether the whole of the calculus has been removed by Heurteloup's instrument, is there anything very grievous in asking less celerity of Professor Dugas in his *first* case? We made no insinuation of falsehood; we insinuated that more time would not have injured the claims of Professor Dugas, and if we ask nothing more of that gentleman than Malgaigne and Curling say is necessary in their cases, in order to give positive opinions, what wrong is done to Professor Dugas? The insinuation that Professor Dugas was "too hasty in his conclusions," and the "insinuation of falsehood," are widely different matters. What possible personal feeling can we have against Professor Dugas? That the whole case is an extraordinary one, Dr. F. must admit, for Professor Dugas claims extraordinary features for it, and it is the nature of extraordinary things to cause surprise. We can see no right that Dr. F. has to ask us not to be surprised at the extraordinary features of Professor Dugas' case of calculus.

Dr. F. objects to an expression we used in saying the patient "heard something drop," and says those words nowhere occur in the report. And pray Dr. who said they did? We purposely used a provincialism, common in Kentucky as expressive of a thing unexpected, and astounding, and on account of its being a provincialism we marked it with inverted commas. In the same paragraph where this phrase occurs, when expressing the language of the patient, we use a term expressive of *feeling* a sensation of something falling.

The comments of Dr. F. upon the distinctions between "just an inch" and "about an inch" are rather small. The use of just instead of about, could have been nothing but an inadvertance, for immediately above our remarks, we had published the full report of Prof. Dugas, and thus furnished our readers the means of correcting any error we might make.

In alluding to our remarks about the dimensions of the calculus of Dr. Dugas's case, Dr. F. says he has seen a stone of the mulberry variety, "the longer or shorter diameters of which, although a little greater, were yet nearly in the same *ratio* with those of the one in question," This stone was removed by Dr. H. F. Campbell. The inference which Dr. F, tries to draw is that this sustains Dr. Dugas's measurement of his oxalate of lime calculus. Dr. F. says; "the Profession demands candor, fair dealing and justice which is proof against all seduction," and we say so too. Dr. F. surely could not expect us to know the measurements of Dr. Campbell's calculus, before he extracted it, and Dr. F. does not pretend that he knows of any other calculi of the proportions named. We had never heard of one before the case of Dr. Dugas, and we said so without expressing any opinion as to the possibility of such a formation. The ratio was named by us as one of the extraordinary features of the case, and it remains so yet.

Can Dr. F. take Heurteloup's crushing instrument, and measure the length of this calculus of Dr. Campbell, in a basin of water, in any position in which he can hold the instrument in the bladder?

We never insinuated aught against the varacity of Prof. Dugas, nor can Dr. F. point to an instance of it. A man may doubt the infallibility even of a Professor's opinions and judgments without implying anything against his veracity. The doubts expressed about the case of Professor Dugas are sustained by lithotomists in whose judgments we rely. We conversed with several before we made any remarks on the case, and each of them took the same view of the matter that we did, and no one of them supposed that he was impugning the veracity of Prof. Dugas.

The medical profession are entitled to clear well ascertained facts in all medical and surgical matters, and no one has a right to complain when extraordinary phenomena and inferences are reported, if they are subjected to rigid examination. This examination is a duty which medical editors owe to the profession at large, and it must happen occasionally that abuse, reviling, and misrepresentation will be the reward of such labors. These are often the only returns the physician gets for his most meritorious services, and the medical editor must look for similar rewards. We have no doubt that if Dr. F. could have used good argumentation, he would have dealt less in personalities.

The medical journals, within the past year, have exhibited some very extraordinary papers, but an editor who fears abuse and denunciation scarcely dares to refer to them except in the way of praise. In the *Medical Examiner*, published in Philadelphia, Dr. Ramsey, of Georgia, sometime since recorded some wonderful statistics, which attracted the attention of the chairman of the committee on obstetrics, of the American Medical Association, and he introduced Dr. Ramsey's statements into his report. When it was made to the Association, Dr. Robinson, of S. C., rose and denounced the statistics of Dr. Ramsey, and avowed himself able to demonstrate their falsity. If Dr. Robinson is right, he certainly deserves thanks for this arrest of false statistics, but we have no doubt that if Dr. Ramsey had had access to a medical journal, Dr. Robinson would have been denounced in the most approved and savage style. Quite recently, a report was published of a case of ovarian tumor, which was relieved of its contents by the introduction of a catheter through the Fallopian tube; but who that values his personal character would hint that it was an extraordinary case, and inexplicable in many of its features? Passion, rage, and violence would fly to the rescue, and talk as oily of professional honor and etiquette as though they belonged to the worst phases of human character.

That the case of Dr. Dugas was a very extraordinary one Dr. F.

must admit, for it has no analogue in the records of medicine. And we asked explanations of some of its features. Instead of giving anything of the kind, Dr. F. enters into a personal warfare, but admits that if he had "patience" he could explain one surprizing part ot the case. Now we think that "patience" was the very virtue that Dr. F. needed, and if he had possessed that christian grace, he might have acquitted himself with more credit, and rendered some service to "the choice between Lithotrity and Lithotomy." We can assure Dr. F. that as the matter stands now, among lithotomists in this region the case is not regarded as a contribution yet to surgical science. The judgment of Malgaigne, Curling and other lithotomists of high authority, stands in the path, and Dr. F's denunciation cannot remove it. Like cases of lithotrity in the hands of the most eminent surgeons in the world, this case must wait the test of time.

The character of Dr. F's response to our "Remarks" is a compliment to the strength of the objections we made in April, and we cheerfully submit that we cannot bandy epithets and personalities with Dr. F. His forte is in those materials; we are content with the less fiery, but more forcible offices of reasoning, argument and truth. B.

